# The effect of A1 and A2 reactive astrocyte expression on hydrocephalus shunt failure

**DOI:** 10.1186/s12987-022-00367-3

**Published:** 2022-09-28

**Authors:** Fatemeh Khodadadei, Rooshan Arshad, Diego M. Morales, Jacob Gluski, Neena I. Marupudi, James P. McAllister, David D. Limbrick, Carolyn A. Harris

**Affiliations:** 1 Department of Chemical Engineering and Materials Science, Wayne State University, Detroit, MI USA; 2 School of Medicine, Wayne State University, Detroit, MI USA; 3 Department of Neurosurgery, Washington University School of Medicine, St. Louis, MO USA; 4 Department of Neurosurgery, Wayne State University School of Medicine, Detroit, MI USA; 5 Department of Biomedical Engineering, Wayne State University, Detroit, MI USA

**Keywords:** Neuroprosthetic device failure, Hydrocephalus, Glial Scar, A1 and A2 reactive astrocyte phenotype, Targeted drug delivery

## Abstract

**Background:**

The composition of tissue obstructing neuroprosthetic devices is largely composed of inflammatory cells with a significant astrocyte component. In a first-of-its-kind study, we profile the astrocyte phenotypes present on hydrocephalus shunts.

**Methods:**

qPCR and RNA in-situ hybridization were used to quantify pro-inflammatory (A1) and anti-inflammatory (A2) reactive astrocyte phenotypes by analyzing C3 and EMP1 genes, respectively. Additionally, CSF cytokine levels were quantified using ELISA. In an in vitro model of astrocyte growth on shunts, different cytokines were used to prevent the activation of resting astrocytes into the A1 and A2 phenotypes. Obstructed and non-obstructed shunts were characterized based on the degree of actual tissue blockage on the shunt surface instead of clinical diagnosis.

**Results:**

The results showed a heterogeneous population of A1 and A2 reactive astrocytes on the shunts with obstructed shunts having a significantly higher proportion of A2 astrocytes compared to non-obstructed shunts. In addition, the pro-A2 cytokine IL-6 inducing proliferation of astrocytes was found at higher concentrations among CSF from obstructed samples. Consequently, in the in vitro model of astrocyte growth on shunts, cytokine neutralizing antibodies were used to prevent activation of resting astrocytes into the A1 and A2 phenotypes which resulted in a significant reduction in both A1 and A2 growth.

**Conclusions:**

Therefore, targeting cytokines involved with astrocyte A1 and A2 activation is a promising intervention aimed to prevent shunt obstruction.

**Supplementary Information:**

The online version contains supplementary material available at 10.1186/s12987-022-00367-3.

## Background

Implantation of foreign materials within the brain initiates a series of reactions, collectively called the foreign body reaction (FBR), which aims to eliminate or isolate the implanted foreign material from the host immune system. Upon implantation of large medical devices such as neuroprosthetics, where elimination is not possible, the FBR continues until the device is barricaded from healthy brain tissue. The initial phase of the FBR is blood-device interactions, which occurs immediately upon implantation caused by vasculature or blood–brain barrier (BBB) disruption. This results in the nonspecific adsorption of blood proteins to the device surface through a thermodynamically driven process to reduce surface energy. In addition to the BBB disruption and influx of serum proteins, the immune system is also activated by signals of host cell injury and extracellular matrix (ECM) breakdown proteins, namely fibrinogen and fibronectin adhesion to the device surface. Microglia, the resident immune cells of the central nervous system (CNS) and blood-derived macrophages recognize the protein signals through receptor-mediated pathways such as toll like receptors (TLRs). Ligand binding to TLRs leads to activation of microglia/macrophages and the secretion of pro-inflammatory cytokines such as TNF-α, IL-1α, and IL-1ß [1, 2]. These very potent signaling molecules are rapidly upregulated in the injured CNS, and are observed at the device-tissue interface corresponding to the location of activated microglia/macrophages and exaggerated astrocytes [2–5]. The effects of TNF-α and IL-1β are strongest on astrocyte activation and proliferation, the key member of the CNS immune response. Reactive astrocytes form a physical barrier known as glial scar, where newly formed and hypertrophic astrocytes overlap and play a beneficial role to prevent injury from spreading to surrounding healthy tissue. However, in relation to its effect on neuroprosthetic implants, the glial scar is considered undesirable because elicits device failure [6, 7]. Collectively, the dominant role of cytokines in orchestrating the dynamic crosstalk among cells and mediating device failure is evident.

A deeper understanding of astrocyte phenotype will lead to a more accurate interpretation of failure in chronically indwelling neuroprosthetics. In a landmark study, Barres and colleagues revealed two significantly different reactive astrocyte phenotype, A1 and A2 [8, 9]. The A1 reactive astrocytes produce large volumes of pro-inflammatory substances and neurotoxin capable of inducing neuronal death. The A2 reactive astrocytes upregulate anti-inflammatory substances and many neurotrophic factors, which promote survival and growth of neurons. The A1 neuroinflammatory astrocytes are induced by NF-κB signaling, whereas the A2 scar-forming, proliferative astrocytes are induced by STAT3-mediated signaling [8, 10]. Since glial scar borders are formed by newly proliferated, elongated astrocytes via STAT3-dependent methods, studies strongly suggest that the A2 reactive astrocyte phenotype is present during glial scar formation [9, 11, 12]. Furthermore, in vivo quiescent astrocytes that contact serum upon injury and BBB disruption, express many of the A2 reactive astrocyte genes [10, 13, 14]. A powerful marker for A1 is the complement component C3, specifically upregulated in A1 reactive astrocytes (but not in resting or A2 reactive astrocytes). C3 is one of the most characteristic and highly upregulated genes in A1 and EMP1 is an A2-specific gene.

In the brain, TNF-α, IL-1α and C1q combined propel resting astrocytes into an A1 reactive state [8]. Co-stimulation with TNF-α and IL-1β propel resting astrocytes into an A2 reactive state with neurosupportive characteristics [15]. In fact, TNF-α and IL-1β modulate the glial scar process by stimulating astrocyte IL-6 secretion [16]. IL-6 primarily activates astrocyte proliferation by a positive feed-forward loop, further activating local astrocytes to maintain the glial scar through self-sustaining mechanisms. IL-6 signaling pathways are enhanced in A2 reactive astrocytes, and STAT3 is activated by IL-6 [10, 17]. This places IL-6 as one of the initial triggers of reactive astrocytes in the acute phase of disease, involved in improving neuronal survival and neurite growth [6, 7]. Although, these properties are evidence of the beneficial roles of IL-6 in repair and modulation of inflammation in the CNS, overproduction of IL-6 is associated with glial scar formation. Inhibition of both IL-6 and IL-6r by antibody neutralization reduces glial scar formation on the implanted device and damage to the brain as a result of bystander effects of increased CSF cytokine levels [18]. Hence, a careful inflammatory balance of IL-6 is essential for proper repair.

Hydrocephalus is a neurologic disorder that results from overproduction of cerebrospinal fluid (CSF) in the ventricles of the brain. The most common treatment paradigm is CSF diversion, typically with insertion of a shunt system. Shunts are unfortunately plagued by high failure rates (40% in the first year, 90% in the first ten years) [20–22], imposing a significant burden on patients, their families, and society. Understanding the root causes of shunt failure will help improve device design and potentially reduce the clinical burden of shunt failure. Shunts fail due to obstruction of the shunt system from adherence of inflammatory cells [24–29]. Our recent work indicates that astrocytes and macrophages are the dominant cell types that bind directly to a ventricular shunt and that astrocytes makeup the vast majority of the cells [30]. We have also found astrocyte markers in obstructive masses to be co-localized with proliferative markers, indicating that the astrocytes were active on the shunt surface; producing inflammatory cytokine IL-6 and proliferating [31]. In hydrocephalus patients, IL-6 cytokines significantly increase during shunt failure upon which astrocyte numbers and reactivity peaks. Astrocytes create a “glue” for more glia or other cells and tissues to secondarily bind and block the shunt. Even shunt contact with the ventricular wall results in astrocyte migration to the surface and interaction with the shunt [32]. Moreover, astrocytes are mechanosensitive, having seen that they produce varying degrees of proliferation inducing IL-6 with different shear/catheter designs [6].

In this first-time study, our goal is to observe whether the cells blocking shunts are expressing an A1 or A2 reactive astrocyte phenotype to understand how to mitigate the cell immune response to shunts. To address this new research avenue, we use qPCR and RNA in-situ hybridization methods. ELISA confirms the pro- and anti-inflammatory cytokine concentration profiles in the CSF associated with astrocyte activation. Then in an in vitro model of astrocyte growth on shunts, we employ neutralizing antibodies to target cytokines involved with astrocyte activation to reduce astrocyte growth. This will keep any attaching astrocytes in a resting state, reduce proliferation, inhibit downstream proliferation, and ultimately deter shunt obstruction. Since the master cytokine IL-1 (α and β) is the initial molecular mediator that triggers glial scar formation around other devices in the brain, we will investigate whether astrocytes obstructing shunts could be prevented by blocking secretion or action of these cytokines to keep astrocytes out of the A1 or A2 reactive state. FDA-approved drugs targeting TNF-α, IL-1α and IL-1β already exist and are in use for other medical conditions.

## Methods

### Ethics approval and sample collection

The permission to collect shunt hardware, CSF, and patient data was approved by the Wayne State Institutional Review Board (IRB) as the coordinating center and as a participating site. Written informed consent was obtained from all patients or their legally authorized representative. Collection was performed in a manner consistent with the standard of treatment: neurosurgeons only removed shunts from patients who presented for symptoms indicative of shunt failure. Samples were collected from individuals with any hydrocephalus etiology and clinical history. After removal by a surgeon, the shunt samples were immediately processed. For RNA in-situ hybridization, samples were first fixed in 4% paraformaldehyde (PFA). Samples were then classified as obstructed/non-obstructed based on the degree of tissue obstruction. According to the manufacturer’s (ACD) instructions a whole-mount procedure was used for non-obstructed samples, while tissue from obstructed samples was carefully removed from the shunt for OCT mounting. For qPCR, samples were processed in RNAlater to preserve RNA quantity/quality. CSF was collected at the time of shunt surgery and transported on ice to the Washington University School of Medicine. Samples were then centrifuged, and the supernatant was stored at − 80 °C until experimental analysis.

### Quantitative PCR (qPCR)

Total RNA was extracted using the GenElute Mammalian Total RNA Miniprep Kit (sigma), cDNA synthesis was performed using the iScript cDNA Synthesis Kit (Bio-Rad), and qPCR was completed using the PowerUp SYBR Green Master Mix (Applied Biosystems) according to manufacturer protocols. Relative mRNA expression was normalized to hRPLP0 (reference gene) [33]. Primers for human are as follows: hC3 (A1 reactive astrocyte marker), hEMP1 (A2 reactive astrocyte marker) [34]. Primers used were: hC3: fwd CCCTGGCTCCACAGTTCTCT, rev CAAGGAGTCCTGCTTGACCG; hEMP1: fwd GTGCTGGCTGTGCATTCTTG, rev CCGTGGTGATACTGCGTTCC; hRPLP0: fwd GAAACTCTGCATTCTCGCTTCC, rev GATGCAACAGTTGGGTAGCCA.

### RNAscope fluorescent in situ hybridization

RNAscope fluorescent in situ hybridization was performed on fixed frozen tissue. Tissue was embedded in OCT compound (Tissue-Tek) and 14 μm tissue sections were prepared and immediately frozen at − 80 °C. Multiplex RNAscope was performed according to manufacturer’s (ACD: Advanced Cell Diagnostics) protocol against the target mRNA probes of hC3 (label for A1 reactive astrocytes), hEMP1 (label for A2 reactive astrocytes), and hSLC1A3 (label for astrocytes). Images were acquired with a resonance-scanning confocal microscopy (RS-G4 upright microscope, Caliber ID, Andover, MA, USA). RNAscope is nonlinearly amplified and thus intensity cannot be used to measure expression. Images were quantified using a threshold in ImageJ. The percent of area above the signal threshold was then quantified and recorded [33].

### Multiplex ELISA

Multiplex assays were run by the Bursky Center for Human Immunology & Immunotherapy Programs Immunomonitoring Laboratory at Washington University School of Medicine. Frozen supernatant CSF was rapidly thawed at 37 °C and centrifuged at 15,000*G* for 5 min prior to analyzing with two multiplex immunoassay kits according to the manufacturer’s instructions for the following inflammatory cytokines: IL-1α, IL-1β, IL-6, TNF-α, IL-8, IL-10 (ThermoFisher Scientific), C3, and C1q (Millipore Sigma). Briefly, magnetic beads and assay buffer were added to all the wells, followed by CSF samples and standards which were added in duplicate. Following washing steps, the detection antibody was added and followed by a streptavidin phycoerythrin incubation. Beads were resuspended with sheath fluid and 50 beads per region were acquired on a Luminex FLEXMAP3D system. The concentration of each analyte was calculated by comparing the sample mean fluorescent intensity to the appropriate standard curve. Belysa v.1 software (Millipore Sigma) was used to generate a 5-parameter logistical curve fit algorithm. Protein concentration is reported as pg/ml for each analyte.

### In vitro model of astrocyte phenotype modulation on shunt material

#### Purification of astrocytes by immunopanning process

Astrocytes were purified by immunopanning from P5 mouse brains and cultured as previously described [35]. Cerebral cortices were dissected and enzymatically digested using papain at 37 °C and 10% CO_2_. Tissue was then mechanically triturated with a serological pipette at RT to generate a single-cell suspension. The suspension was filtered and negatively panned for microglia/macrophage cells (CD45), oligodendrocyte progenitor cells (O4 hybridoma), and endothelial cells (L1) followed by positive panning for astrocyte cells (ITGB5). Astrocytes were cultured in defined, serum-free medium containing 50% neurobasal, 50% DMEM, 100 U/ml penicillin, 100 μg/ml streptomycin, 1 mM sodium pyruvate, 292 μg/ml l-glutamine, 1× SATO, 5 μg/ml of N-acetyl cysteine, and 5 ng/ml HBEGF.

All animal protocols were approved by the Institutional Animal Care and Use Committee at Wayne State University (IACUC).

#### Targeted drug delivery

A1 reactive astrocytes were generated by culturing the purified astrocytes for 6 days in serum free culture on PDMS coated tissue culture plates and then treating for 24 h with IL-1α (3 ng/ml, Sigma, I3901), TNF-α (30 ng/ml, Cell Signaling Technology, 8902SF), and C1q (400 ng/ml, MyBioSource, MBS143105). A2 reactive astrocytes were generated by culturing the purified astrocytes for 6 days in serum free culture on PDMS coated tissue culture plates and then treating for 24 h with IL-1β (30 ng/ml, Cell Signaling Technology, 8900SF) and TNF-α (30 ng/ml, Cell Signaling Technology, 8902SF). Then A1 reactive astrocytes were targeted for 48 h using neutralizing antibodies to IL-1α (30 ng/ml, Abcam, ab9614), TNF-α (30 ng/ml, Cell Signaling Technology, 7321), and TGF-β (30 ng/ml, R&D Systems, 243-B3-002/CF). And A2 reactive astrocytes were targeted for 48 h using neutralizing antibodies to IL-1β (30 ng/ml, Abcam, ab9722), TNF-α (30 ng/ml, Cell Signaling Technology, 7321), and IL-6 (30 ng/ml, Abcam, ab6672) [8, 36]. Since the astrocytes are growing on PDMS coated tissue culture plates in serum free conditions they reach about 50% confluency after 6 days in the 24-well plate. Images of the 24-well plate was acquired with a Zeiss Oberver.Z1 microscope. Cell count covering each well of a 24-well plate with a surface area of 1.9 cm^2^ was measured using ImageJ.

Polydimethylsiloxane (PDMS) coated tissue culture plates were prepared by mixing Sylgard-184 elastomer and curing agents at a ratio of 10:1 (w/v), then pouring into the plates and curing for 48 h at RT.

### Data presentation and statistical analysis

Data analysis was performed using GraphPad Prism version 8 software. All data are presented as mean ± standard error of the mean (SEM). Shapiro–Wilk test was performed to examine if the data were normally distributed. All normally distributed data sets were analyzed using parametric tests (two-tailed unpaired Student’s t-test and two-way ANOVA). Non-normal data sets were analyzed using a non-parametric test (Mann Whitney U test). All p values below 5% were considered statistically significant; *p < 0.05.

## Results

### Quantifying reactive astrocyte genes on collected tissue from failed shunts

**Fig. 1:**
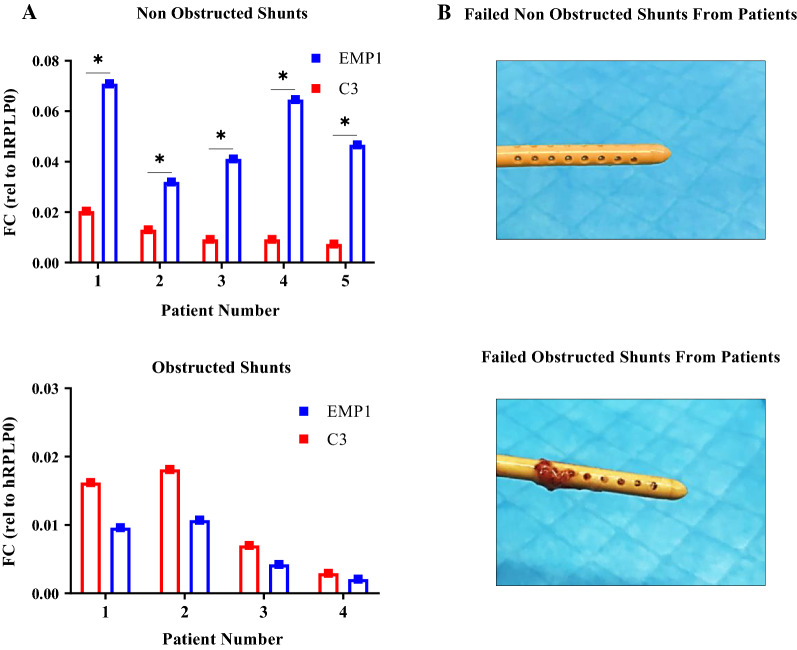
Expression of C3, EMP1 astrocyte activation genes assessed by qPCR on obstructed and non-obstructed shunts. **A** Comparing the expression of the A2-specific gene EMP1 and the A1-specific gene C3 to the housekeeper gene hRPLP0 in every single patient for obstructed and non-obstructed shunts. Statistical analysis for fold changes was carried out using two-way ANOVA; *p < 0.05. **B** Representative images for obstructed and non-obstructed shunts collected from patients. Obstructed and non-obstructed shunts were characterized based on the degree of actual tissue blockage on the shunt surface instead of clinical diagnose

qPCR results demonstrated a heterogeneous upregulation of both the A1- and the A2-specific reactive gene on both obstructed and non-obstructed shunts. The A2-specific gene EMP1 trended towards significantly higher expression on non-obstructed shunts (two-way ANOVA; p = 0.0381, Fig. 1), while the A1-specific gene C3 trended towards higher expression on obstructed shunts due to persistent neuroinflammation, however, the trend was not significant. The qPCR results are not specific to astrocyte cells but all the cells on the shunt surface.

### Quantifying reactive astrocyte genes expressed by astrocytes on failed shunts

**Fig. 2:**
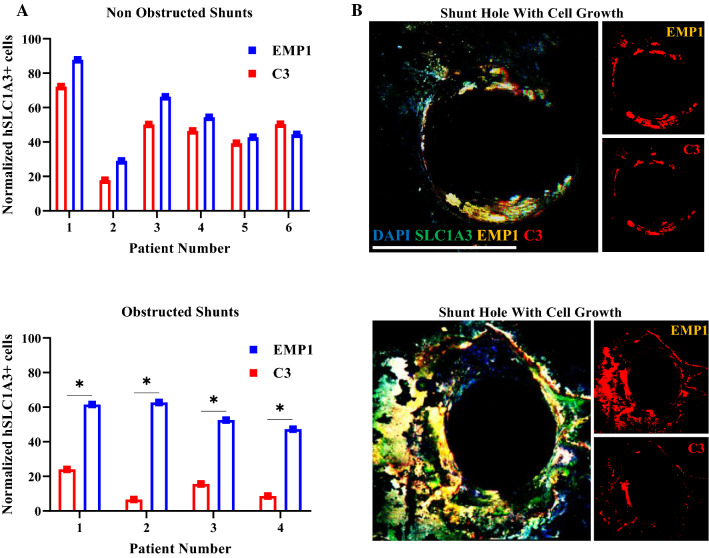
Expression of C3, EMP1 astrocyte activation genes assessed by RNAscope fluorescent in situ hybridization on obstructed and non-obstructed shunts. **A** SLC1A3 was used as an astrocyte marker to confirm that C3 and EMP1 signal represented the A1 and A2 astrocytes specifically. Data for obstructed and non-obstructed shunts are shown. For normalization, the C3 and EMP1 signals were dividing by SLC1A3 signals. Statistical analysis was carried out using two-way ANOVA; *p < 0.05. **B** Representative RNAscope fluorescent in situ hybridization images for obstructed and non-obstructed astrocyte gene C3 (red) and EMP1(yellow) showing colocalization with the astrocyte marker SLC1A3 (green). Separated channels for C3 and EMP1 for each condition are also presented (scale bar = 500 μm)

RNA in-situ hybridization results concurred with qPCR data in finding a heterogeneity in the populations existing in both shunts. A greater number of SLC1A3+ astrocytes expressed the A2-specific gene EMP1 on both obstructed and non-obstructed shunts. Interestingly, the number of A2 reactive astrocytes are significantly greater on obstructed shunts compared to A1 reactive astrocytes (two-way ANOVA; p = 0.0172, Fig. 2). Moreover, as shown in Fig. 2B with separated channels for C3 and EMP1, non-obstructed shunts show more colocalization of A1 and A2 compared to obstructed shunts. The RNA in-situ hybridization results are specific to astrocyte cells on the shunt surface.

### Cerebrospinal fluid biomarkers of neuroinflammation for failed shunts

**Fig. 3:**
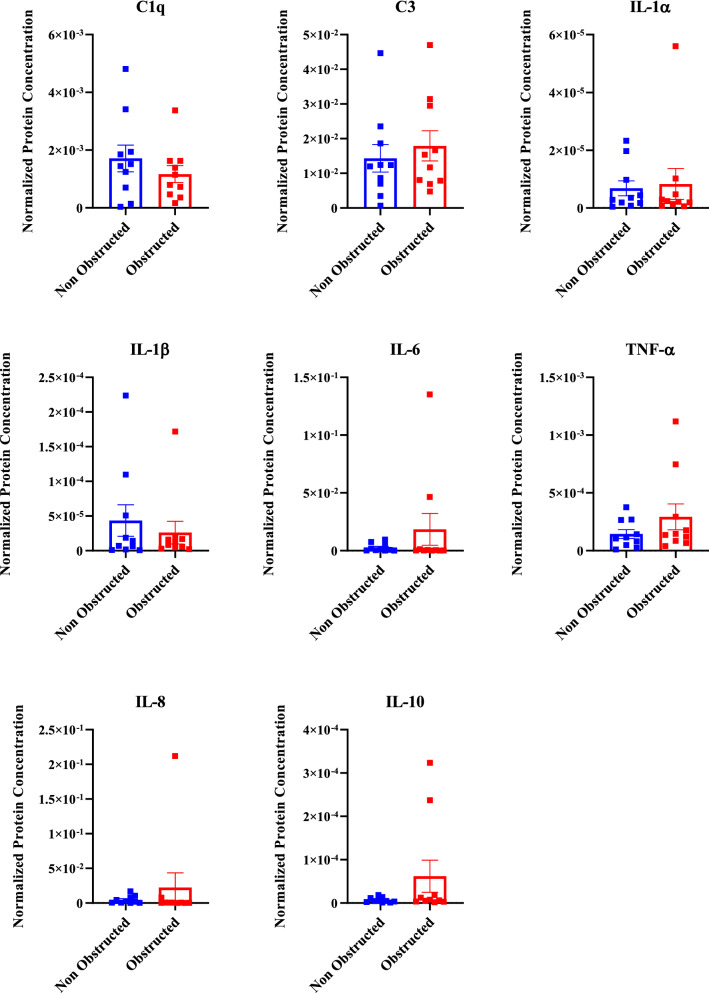
Cerebrospinal fluid cytokine concentrations for obstructed and non-obstructed shunts. Analytes include C3, C1q, and IL-1α (A1 astrocyte markers), IL-1β and IL-6 (A2 astrocyte markers), TNF-α, IL-8 and IL-10. For normalization, the concentration of each cytokine is divided by the total protein concentration for each group. Statistical analysis was carried out using Mann Whitney U test; p > 0.05 (n = 10 per group, mean ± SEM)

ELISA results concurred with qPCR data in finding higher inflammatory cytokine levels for obstructed shunts, however, this was not significantly different from non-obstructed shunts confirming the heterogeneous population of A1 and A2 reactive astrocytes exist on both shut classifications (Mann Whitney U test; p > 0.05, Fig. 3). Interestingly, ELISA results also concurred with RNA in-situ hybridization data in finding that IL-6 trended towards higher levels for obstructed shunts compared to non-obstructed shunts. IL-6 primarily activates A2 reactive astrocyte proliferation through a positive feed-forward loop, activating local astrocytes to maintain the glial scar formation on the shunt surface.

### Inhibiting astrocyte cell activation and adhesion on shunt material with neutralizing antibody treatment and anti-inflammatory cytokines

**Fig. 4:**
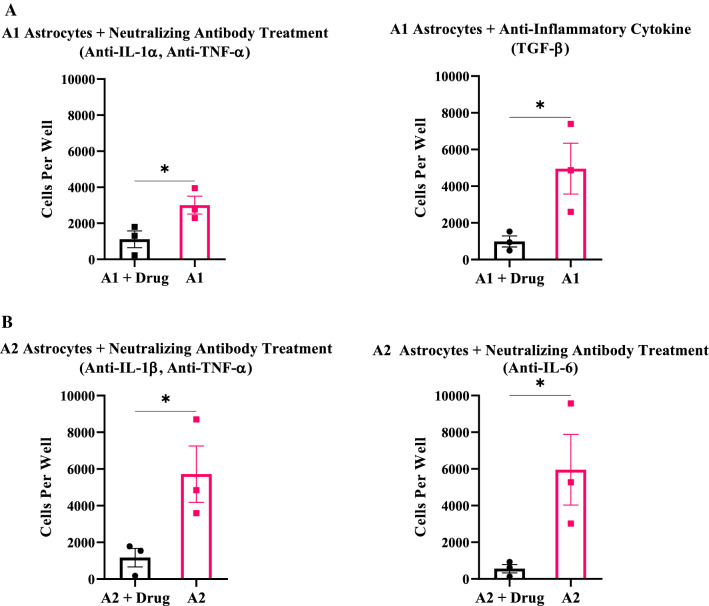
Targeted therapies that inhibit cell activation to reduce adhesion on the shunt surface. **A** A1 reactive astrocytes treated with neutralizing antibodies to TNF-α, IL-1α, and anti-inflammatory cytokine TGF-β. **B** A2 reactive astrocytes treated with neutralizing antibodies to TNF-α, IL-1β, and IL-6. Statistical analysis was carried out using two-tailed unpaired Student’s t-test; *p < 0.05 (n = 3 per group, mean ± SEM). Cell count covering each well of a 24-well plate with a surface area of 1.9 cm^2^ is measured

Given that the first two experiments demonstrated a heterogeneous population of A1 and A2 reactive phenotypes on the shunt surface, we investigated whether the activity of astrocytes could be significantly reduced by employing antibody therapies to inhibit TNF-α, IL-1α, IL-1β, and IL-6. These neutralizing antibodies were chosen to decrease the activity of A1 and A2 astrocytes with the aim of significantly decreasing adhesion on PDMS coated surfaces mimicking the shunt surface. The anti-inflammatory cytokine TGF-ß was also able to decrease the activity of A1 astrocytes, significantly reducing cell adhesion on the PDMS coated surface (two-tailed unpaired Student’s t-test; p < 0.05, Fig. 4) (Additional file 1).

## Discussion

This first-time study, which pulls strength from the recent landmark Barres et al. study on astrocyte activation, presents a robust investigation of the changes in gene expression levels specific to astrocyte immune response following CSF shunt implantation. By shedding light on the mystery of astrocyte phenotype expression on shunt surfaces, our understanding of the root causes for shunt failure is improved to revolutionize hydrocephalus treatment paradigms.

qPCR and RNA in-situ hybridization results demonstrated a heterogeneous upregulation of both the A1- and the A2-specific reactive gene on both obstructed and non-obstructed shunts. Based on qPCR results the A2-specific gene EMP1 trended towards higher expression on non-obstructed shunts. Astrocytes are the dominant cell type bound directly to non-obstructed shunts and play a neuroprotective role, particularly in the acute phase of injury following an immediate disruption of the blood–brain barrier (BBB). Therefore, the increased expression of EMP1 on non-obstructed shunts is in accordance with other studies [28]. Furthermore, based on qPCR results the A1-specific gene C3 trended towards higher expression on obstructed shunts. Indeed, A1 reactive astrocytes are a major source of the classical complement cascade component C3, however, other inflammatory cells in the tissue on obstructed shunts also induce the expression of C3. The increased expression of C3 on obstructed shunts is in accordance with other studies linking persistent neuroinflammation to neurodegeneration and adverse effects on the neural circuits and decrease excitatory neuronal function [37]. This is to recruit additional immunocytes to the site and exacerbate the secondary insult response. Therefore, suppressing A2 activation even in A1 and A2 colocalized cells, will not exacerbate A1 pathway activation in those astrocytes and therefore will not increase neural death around the implant since we are attenuating persistent inflammation.

Based on RNA in-situ hybridization results the number of A2 reactive astrocytes are significantly greater on obstructed shunts compared to A1 reactive astrocytes. Since A2 reactive astrocytes are proliferative [9], they are considered to be responsible for glial scar formation observed on obstructed shunts. In our recent work, we have also observed astrocyte markers in obstructive masses to be co-localized with proliferative markers, indicating that astrocytes are active on the shunt surface as they produce inflammatory cytokine IL-6 and proliferate [31]. This is in accordance with other studies, indicating that glial scar borders are formed by newly proliferated, elongated astrocytes that interact to corral inflammatory and fibrotic cells via STAT3-dependent mechanisms [12], and that astrocytes in scar formation seem to be devoid of C3 upregulation [38]. In our recent paper, higher levels of IL-6 are observed for non-obstructed shunts compared to obstructed shunts [39]. However, obstructed and non-obstructed shunts were characterized based on the patient’s symptoms and clinical presentation as evaluated by the neurosurgeon, instead of the degree of actual tissue blockage on the shunt surface as described in this study. Classifying shut obstruction based on real obstruction instead of clinical diagnose is an important improvement with respect to other studies. The dominance of A2 on obstructed shunts does not insinuate that there is no subgroups or activation states dependent on CSF environment, but instead that we can manipulate the cytokine levels in the microenvironment around the shunt which could have a specific impact on astrocyte phenotype.

The cytokines for ELISA studies were picked with intention based on our recent paper [39] that observed CSF concentrations dependent on obstruction of IL-10, IL-6, IL-8.

Our targeted therapy results of inhibition of astrocyte cell activation mean less overall proliferation and/or attachment in the neutralizing conditions. In other studies, we have observed that A1 and A2 cells will increase their attachment following increased concentrations of cytokine [31]. For example, A2 cells with more IL-6 significantly increases their growth, suggesting a more proliferation dependency than death. If cell death is occurring, we suspect it is only a result of the cells inability to attach. The neutralizing antibodies are against the cytokines which promote astrocytes to fall into general categories of reactive complement (A1) or reactive neurotrophic (A2) astrocyte cascades. If inhibiting one or the other, any of these neutralizing antibodies would also decrease the number of astrocytes possibly expressing A1 and A2 simultaneously. Since we imagine engineering strategies where neutralizing antibodies will be attached to the surface of the shunt and/or released at a very low concentration, it is our intent that any impact would be localized without it influencing any astrocytes in the parenchyma. Of course, safety data would need to be exhaustive in this manner. In future work we could also compare to other works where we look at morphology with glial fibrillary acidic protein (GFAP) alongside counting A1 and A2 astrocytes. This proof-of-concept study to see if neutralizing antibodies could be effective may have a different effect on colocalization of A1 and A2 in same cells vs A2 cells vs A1 cells. We will explore the dose and time dependency in future work in a more physiologic system to understand its role on shunt function and viability of periventricular tissue. Dose and time dependency is important since colocalization likely occurs if the cell is transitioning its reactive state because of a change in CSF microenvironment. Concentration and release rate of neutralizing antibodies would minimize the number of A1 or A2 cells, and indirectly minimize the number of those expressing A1 and A2 simultaneously (we would neutralize the cytokine that exacerbates cells in an A1 or A2 phenotype, even if there are subgroups within the A1 and A2 classification).

Targeted therapies that inhibit astrocyte cell activation were able to significantly reduce astrocyte cell adhesion on PDMS coated surfaces mimicking the shunt surface. These results are in accordance with other studies indicating that the knockout of reactive astrocyte activating factors slows disease progression [33], dampening the formation of reactive astrocytes prevents neuronal death [36], and astrogliosis inhibition attenuates hydrocephalus [40, 41]. TGF-ß suppresses A1 astrocyte activation [8], reverses the formation of A1 astrocytes by fibroblast growth factor (FGF) signaling [42], and greatly reduces the expression of A1-specific markers [43].

In extensive studies, chronically implanted neural implants with coatings were compared to that of identical uncoated devices. The coated implant significantly reduced astrocyte and microglial adhesion [44, 45]. However, no significant difference was observed in the neuroinflammatory response or the level of neuronal loss surrounding the coated implant compared to uncoated devices. Our recent paper also indicates that under higher shear stress, despite less astrocyte cell adhesion to the surface, a significant increase in IL-6 secretion is detected [31]. Our data in combination with previous studies, identify that to have maximal impact procedures should implement a focus on attenuating the initial inflammatory cell activation instead of only focusing on reducing cell adhesion on the device surface in vivo [4, 6, 45–46]. Procedures such as decreasing shear activation of cells [2, 4, 6, 7, 48–51], and directly antagonizing the accumulation of pro-inflammatory cytokines via targeted therapeutic for TNF-α, IL-1α, Il-1β, and IL-6.

Collectively, the data suggest that drug therapies could be added to the neuroprosthetic device as coating and released in vivo for enhanced device performance within the brain. Cytokine responses were strongly upregulated within minutes of implantation indicating cytokine targeting strategies need to be introduced at the site of implantation concurrent with or immediately following implantation. However, Short-term delivery of anti-cytokine therapies are incapable of attenuating astrocyte activation and adhesion over extended periods of time. To develop a system to provide persistent protection, initial burst release followed by constant demonstration of an immobilized layer of the neutralizing antibody on the shunt surface must be utilized. Based on our results, since both A1 and A2 reactive astrocytes are present for non-obstructed shunts, we could initially engineer a burst release of neutralizing antibodies to attenuate both A1 and A2 activation. If colocalized, inhibition of TNF-α may inhibit both A1 and A2, which can be explored in future work. Then, since A2 reactive astrocytes are significantly greater on obstructed shunts, we could engineer an immobilized layer of neutralizing antibody on the shunt surface to be released at a very low concentration to attenuate A2 activation for persistent and localized protection. However, in cases where the immobilization process changes the properties of the neutralizing antibodies, exosomes, nanoparticles produced by almost all cells, are utilized. Exosomes are increasingly employed as therapeutic delivery vehicles to enhance CNS diseases as they can cross the blood–brain barrier and directly target cells over long distances with low immunogenicity.

Overall, these data point to the necessity to understand the function of A1 and A2 reactive astrocytes on shunts in the treatment of hydrocephalus. However, there are other subgroups of astrocytes [52] and dividing by subgroups (as macrophages) is an important next step in really understanding mechanisms of astrocyte activity on shunts. Importantly, obstructive shunt failure can occur as a complication of mispositioning or migration of the shunt in close contact with the choroid plexus and deserves its own in-depth study. If tissue is contacting the shunt, is failure occurring from cells/tissue getting physically pulled in, cells growing in, or a combination of both? Is this caused from single or repetitive contact? Calculating the daily cellular passage through the shunt would identify one source of cells (cells that have shed from ependyma which reveals reactive astrocytes in the periventricular tissue), which is a great additional study.

For a more conclusive work future qPCR and RNA in-situ hybridization studies must analyze other markers of reactive astrocytes such as cytoskeletal, metabolic, signaling, ion channels, etc. Likewise, future ELISA studies must further analyze other arrays of different cytokines. For a more relevant in vivo situation future studies must also include other cell types such as macrophages and immune cells and include implantation of the device in the mouse brain and then explore astrogliosis around the device in the absence and presence of various neutralizing antibodies or receptor antagonists.

## Conclusion

This novel study improves our understanding of shunt failure and the underlying astrocyte phenotype expression profiles modulating these failures. This work presents a better understanding of cellular response mechanisms to improve device implantation and a direction for future researchers to extend the life of chronically indwelling neuroprosthetics. Additionally, this work narrows the therapeutic window to effectively inhibit astrocyte activation and adhesion on implants. Furthermore, we suggest that for significant reduction in shunt failure, manipulating shunt physical properties such as shear stress along the shunt/CSF interface as well as drug therapies added to the device as coating to inhibit cytokines and cell aggregation is necessary. A combinatorial strategy will lead to cumulative improvements in CSF shunt technology and improve clinical outcomes to reduce disease burden, healthcare costs and improve the quality of life for hydrocephalus patients.

## Supplementary Information

**Additional file 1: Fig. S1.** Microglia/macrophage and astrocyte reactions following neuroprosthetic device implantation. Injury transforms microglia into an M1- and M2-like phenotype and astrocytes into an A1- and A2-type, correspondingly. Astrocytes and microglia work together to initiate either a neuroinflammatory or neuroprotective response after injury through the release of cytokines or neurotrophic factors that can lead to neuronal death or survival. The cytokine pathway is the most important measurable outcome for inflammatory cascades. Inflammatory cells at the brain-device interface communicate via cytokines to activate and recruit other inflammatory cells to the interface. Cytokine stimulation is the gateway for other gene products to be over- or under-expressed in the cascade, resulting in device failure. Therefore, the cytokine pathway acts as a starting point for mechanistic, thorough investigation of inflammation and device failure. **Table S1.** Ct values for patients with non-obstructed and obstructed shunts.

## Data Availability

All data generated/analyzed during this study are included in this published article. All other relevant data that support the findings of this study are available from the corresponding author upon reasonable request. Data are currently stored locally and approved cloud services at Wayne State University.

## Abbreviations

A1: Astrocyte cell neuroinflammatory reactive phenotype
A2: Astrocyte cell scar-forming, proliferative reactive phenotype
BBB: Blood–brain barrier
C1q: Complement component 1q
C3: Complement component 3
CNS: Central nervous system
CSF: Cerebrospinal Fluid
ECM: Extracellular matrix
ELISA: Enzyme-linked immunoassay
EMP1: Epithelial membrane protein 1
FBR: Foreign body response (neuroinflammation to device)
FDA: Food and Drug Administration
IL-1α: Interleukin 1 alpha (pro-inflammatory cytokine)
IL-1β: Interleukin 1 beta (pro-inflammatory cytokine)
IL-6: Interleukin 6 (anti-inflammatory cytokine)
M1: Microglia/macrophage cell pro-inflammatory phenotype
M2: Microglia/macrophage cell immunosuppressive phenotype
NF κB: Nuclear factor kappa B
PDMS: Polydimethylsiloxane
STAT3: Signal transducer and activator of transcription 3
TGF-β: Transforming growth factor beta (anti-inflammatory cytokine)
TLR: Toll like receptor
TNF-α: Tumor necrosis factor alpha (pro-inflammatory cytokine)
