# Supplementary material for: The effect of A1 and A2 reactive astrocyte expression on hydrocephalus shunt failure
Source: Fluids Barriers CNS. 2022 Sep 28;19:78. doi: 10.1186/s12987-022-00367-3 (PMC9516791; doi:10.1186/s12987-022-00367-3)
Supplement: Supplementary file 1 — Additional file 1: Fig. S1. Microglia/macrophage and astrocyte reactions following neuroprosthetic device implantation. Injury transforms microglia into an M1- and M2-like phenotype and astrocytes into an A1- and A2-type, correspondingly. Astrocytes and microglia work together to initiate either a neuroinflammatory or neuroprotective response after injury through the release of cytokines or neurotrophic factors that can lead to neuronal death or survival. The cytokine pathway is the most important measurable outcome for inflammatory cascades. Inflammatory cells at the brain-device interface communicate via cytokines to activate and recruit other inflammatory cells to the interface. Cytokine stimulation is the gateway for other gene products to be over- or under-expressed in the cascade, resulting in device failure. Therefore, the cytokine pathway acts as a starting point for mechanistic, thorough investigation of inflammation and device failure. Table S1. Ct values for patients with non-obstructed and obstructed shunts. [file 12987_2022_367_MOESM1_ESM.docx]

**Fig. S1.** Microglia/macrophage and astrocyte reactions following neuroprothetic device implantation. Injury transforms microglia into an M1- and M2-like phenotype and astrocytes into an A1- and A2-type, correspondingly. Astrocytes and microglia work together to initiate either a neuroinflammatory or neuroprotective response after injury through the release of cytokines or neurotrophic factors that can lead to neuronal death or survival. The cytokine pathway is the most important measurable outcome for inflammatory cascades. Inflammatory cells at the brain-device interface communicate via cytokines to activate and recruit other inflammatory cells to the interface. Cytokine stimulation is the gateway for other gene products to be over- or under-expressed in the cascade, resulting in device failure. Therefore, the cytokine pathway acts as a starting point for mechanistic, thorough investigation of inflammation and device failure.

**Table S1.** Ct values for patients with non-obstructed and obstructed shunts.

| **Non-Obstructed Shunts Ct Values** | | | | | |
| --- | --- | --- | --- | --- | --- |
| Patient # | 1 | 2 | 3 | 4 | 5 |
| hRPLP0 | 21.154 | 21.422 | 19.359 | 19.477 | 21.242 |
| C3 | 26.768 | 27.686 | 26.117 | 26.238 | 28.328 |
| EMP1 | 24.971 | 26.390 | 23.965 | 23.428 | 25.663 |

| **Obstructed Shunts Ct Values** | | | | |
| --- | --- | --- | --- | --- |
| Patient # | 1 | 2 | 3 | 4 |
| hRPLP0 | 17.016 | 17.554 | 17.425 | 17.758 |
| C3 | 22.963 | 23.341 | 24.581 | 26.167 |
| EMP1 | 23.717 | 24.112 | 25.306 | 26.654 |
